# Alterations in Cerebral White Matter and Neuropsychology in Patients with Cirrhosis and Falls

**DOI:** 10.1371/journal.pone.0118930

**Published:** 2015-03-20

**Authors:** Beatriz Gómez-Ansón, Eva Román, Ramón Fernández de Bobadilla, Patricia Pires-Encuentra, Jordi Díaz-Manera, Fidel Núñez, Saül Martinez-Horta, Yolanda Vives-Gilabert, Javier Pagonabarraga, Jaume Kulisevsky, Juan Cordoba, Carlos Guarner, Germán Soriano

**Affiliations:** 1 Neuroradiology Unit, Department of Radiology, Hospital de la Santa Creu i Sant Pau, Barcelona, Spain; 2 Department of Gastroenterology, Hospital de la Santa Creu i Sant Pau, Barcelona, Spain; 3 Movement Disorders Unit, Department of Neurology, Hospital de la Santa Creu i Sant Pau, Barcelona, Spain; 4 Neuromuscular Disorders Unit, Department of Neurology, Hospital de la Santa Creu i Sant Pau, Barcelona, Spain; 5 Institut de Recerca IIB-Sant Pau, Hospital de la Santa Creu i Sant Pau, Barcelona, Spain; 6 Escola Universitària d’Infermeria EUI-Sant Pau, Hospital de la Santa Creu i Sant Pau, Barcelona, Spain; 7 Department of Internal Medicine, Hospital Vall d’Hebron, Barcelona, Spain; 8 Port d’Informació Científica (PIC), Universitat Autònoma de Barcelona, Bellaterra (Cerdanyola del Vallès), Spain; 9 Institut de Física d’Altes Energies (IFAE), Universitat Autònoma de Barcelona, Bellaterra (Cerdanyola del Vallès), Spain; 10 Universitat Autònoma de Barcelona, Bellaterra (Cerdanyola del Vallès), Spain; 11 Centro de Investigación Biomédica en Red de Enfermedades Hepáticas y Digestivas (CIBEREHD), Barcelona, Spain; 12 Centro de Investigación Biomédica en Red de Enfermedades Raras (CIBERER), Valencia, Spain; 13 Centro de Investigación Biomédica en Red Enfermedades Neurodegenerativas (CIBERNED), Madrid, Spain; 14 INNDACYT, CR, Sant Feliu de Llobregat, Spain; Charité University Medicine Berlin, GERMANY

## Abstract

**Background & Aim:**

Falls are frequent in patients with cirrhosis but underlying mechanisms are unknown. The aim was to determine the neuropsychological, neurological and brain alterations using magnetic resonance-diffusion tensor imaging (MR-DTI) in cirrhotic patients with falls.

**Patients and methods:**

Twelve patients with cirrhosis and falls in the previous year were compared to 9 cirrhotic patients without falls. A comprehensive neuropsychological and neurological evaluation of variables that may predispose to falls included: the Mini-Mental State Examination, Psychometric Hepatic Encephalopathy Score (PHES), Parkinson’s Disease-Cognitive Rating Scale, specific tests to explore various cognitive domains, Unified Parkinson’s Disease Rating Scale to evaluate parkinsonism, scales for ataxia and muscular strength, and electroneurography. High-field MR (3T) including DTI and structural sequences was performed in all patients.

**Results:**

The main neuropsychological findings were impairment in PHES (p = 0.03), Parkinson’s Disease-Cognitive Rating Scale (p = 0.04) and in executive (p<0.05) and visuospatial-visuoconstructive functions (p<0.05) in patients with falls compared to those without. There were no statistical differences between the two groups in the neurological evaluation or in the visual assessment of MRI. MR-DTI showed alterations in white matter integrity in patients with falls compared to those without falls (p<0.05), with local maxima in the superior longitudinal fasciculus and corticospinal tract. These alterations were independent of PHES as a covariate and correlated with executive dysfunction (p<0.05).

**Conclusions:**

With the limitation of the small sample size, our results suggest that patients with cirrhosis and falls present alterations in brain white matter tracts related to executive dysfunction. These alterations are independent of PHES impairment.

## Introduction

Minimal hepatic encephalopathy is the subclinical cognitive impairment caused by liver insufficiency and portal-systemic shunting that affects at least one third of patients with cirrhosis [1,2]. This disorder predisposes to overt hepatic encephalopathy [1], death [3], deterioration in health-related quality of life [4], and traffic accidents [5]. Cognitive dysfunction in patients with cirrhosis, however, does not necessarily results from minimal hepatic encephalopathy alone. Indeed, it is frequently multifactorial, due to the contribution of other conditions, such as malnutrition, alcohol, hepatitis C virus, diabetes or treatment with psychoactive drugs [6].

A high incidence of falls has been reported in patients with cirrhosis, mainly in those having cognitive dysfunction as determined by the Psychometric Hepatic Encephalopathy Score (PHES) [6], a test battery widely used to diagnose minimal hepatic encephalopathy [1,2]. As in other debilitating disorders [7–10], falls in patients with cirrhosis are associated with fractures, surgery, disease decompensation and mortality [6,11,12]. Moreover, falls are linked to impairment in health-related quality of life [4] and represent a healthcare and social burden for the community [8].

The exact mechanisms underlying falls in patients with cirrhosis are unknown [6,13]. Potential mechanisms include specific cognitive deficits, parkinsonism, impairment in muscular strength, and peripheral neuropathy. All these conditions have been associated with falls in other populations [7–10,14], and are also frequently observed in patients with cirrhosis [2,15–17]. Alterations in cerebral white matter have recently been observed in patients with cirrhosis, mainly in those with cognitive dysfunction [18–20], and they have been related to falls in other populations [21]. Magnetic resonance-diffusion tensor imaging (MR-DTI) allows a precise, non-invasive study of cerebral white matter integrity *in vivo* [22,23]. Identifying the mechanisms underlying the predisposition of patients with cirrhosis to fall could help to establish appropriate preventive measures.

The aim was to determine the neuropsychological, neurological and brain alterations using magnetic resonance-diffusion tensor imaging (MR-DTI) in cirrhotic patients with falls.

## Patients and Methods

### Patient selection

We included 30 outpatients with cirrhosis: 15 with at least one fall during the previous 12 months, and 15 without falls. Patients were selected consecutively from the outpatients visit of the Department of Gastroenterology in Hospital de la Santa Creu i Sant Pau, Barcelona, Spain.

Cirrhosis was diagnosed from clinical, analytical and ultrasonographic findings, or by liver biopsy. Exclusion criteria were: any hospitalization in the previous month due to decompensation of cirrhosis, severe live failure (Model for End-Stage Liver Disease [MELD] score >25), hepatocellular carcinoma or other neoplasia, active alcohol intake (in the previous 12 months), current overt acute or chronic hepatic encephalopathy, neurological disease, marked symptomatic comorbidities, current treatment with psychoactive drugs, and life expectancy less than 6 months. Patients who did not complete all the assessment procedures specified below and those who developed complications of cirrhosis during the study were excluded from the analysis of the results.

We recorded clinical and analytical data, including parameters that influence the predisposition to fall in populations other than cirrhotic patients. These parameters included serum sodium, mean arterial pressure and orthostatic hypotension, pharmacological treatment, body mass index, degree of comorbidity and visual acuity [8,24].

### Falls

We determined falls using the World Health Organization definition: “A fall is an event which results in a person coming to rest inadvertently on the ground or floor or other lower level” [25]. The incidence and characteristics of falls were determined through interviews with the patients and relatives and review of patients’ medical records. We also recorded the severity of injuries and the healthcare needed due to falls [6].

### Neuromuscular evaluation

We performed a complete clinical examination of the nervous system in all patients. This included muscle strength (using the Medical Research Council [MRC] scale) [26], muscle tone, sensory system (light touch, pinprick and vibratory sensation), muscle stretch reflexes, cutaneous-plantar response and cerebellar and brain-stem function (finger to nose and heel to knee maneuvers, oculomotricity, gait and Romberg test).

All patients also underwent an electroneurography that included evaluation of sensory and motor nerves. We explored sural and peroneal nerves first. If no any abnormality was identified, a peripheral neuropathy was ruled out. However, if the results of the evoked sensory or motor potentials were abnormal, the electroneurography was continued and radial sensory evoked potentials and ulnar motor and sensory evoked potentials were examined.

The presence of parkinsonism was assessed using the Unified Parkinson's Disease Rating Scale (UPDRS). Part III of the UPDRS evaluates the motor impact of parkinsonian symptoms [27]. The axial subscore of the UPDRS-III, that assesses axial parkinsonian symptoms, is obtained by the addition of the six items of the UPDRS-III that specifically measure speech, neck rigidity, chair rise, posture, gait, and postural stability [28]. Ataxia and cerebellar dysfunction were assessed using the International Cooperative Ataxia Rating Scale (ICARS) part I (posture and gait evaluation) and part II (kinetic score) [29].

### Neuropsychological assessment

Neuropsychological evaluation included three cognitive scales and several individual tasks to assess specific cognitive domains: attention, memory, language, executive function and visuospatial skills directed to quantify fronto-subcortical and posterior-cortical functions.

The Mini Mental State Examination (MMSE) [30] is the most commonly used instrument for screening cognitive function. The Psychometric Hepatic Encephalopathy Score (PHES) includes a neuropsychological battery used to diagnose minimal hepatic encephalopathy [1,2,31]. We used the computer programme of the Red Española de Encefalopatía Hepática (http://www.redeh.org) [31]. The Parkinson’s Disease-Cognitive Rating Scale (PD-CRS) [32] was mainly designed to capture the whole spectrum of cognitive functions impaired over the course of Parkinson’s disease.

To focus on the specific cognitive domains, we selected the following tests from the extensive compendium of classical neuropsychological tasks: the Forward and Backward Digit Span from the Weschler Memory Scale-3^rd^ edition for attention and working-memory; Verbal Phonetic and Semantic Fluencies for language and executive functions; the Rey-Osterrieth ComplexFigure Test for visuoconstructive skills, visual memory and executive functions; and the Wisconsin Card Sorting Test (WCST) and Iowa Gambling Task, both for executive functions [33].

### Magnetic resonance imaging (MRI)

MRI scans were acquired using a 3 Tesla Philips-Achieva MRI (software version 2.1.3.2). Two neuroradiologists blindly assessed different findings, including white matter hyperintensities on conventional MRI (FLAIR sequences).

#### MR-DTI analysis

DTI was obtained using an echo-planar, DT_SSh_iso, SENSE sequence, (b-value = 800 s/mm², echo time = 60 ms, repetition time = 6672 ms, slice thickness = 2 mm, voxel dimensions = 1.64 x 1,64 x 2 mm, FOV = 100 mm, acquisition matrix = 112 x 112).

DTI processing was performed at the cluster facilities of Port d’Informació Científica, Universitat Autònoma de Barcelona. Images were analyzed with the FMRIB Software Library (FSL, http://www.fmrib.ox.ac.uk/fsl) [34], including eddy current correction with affine registration, and brain extraction. Fractional anisotropy (FA), mean diffusivity (MD), axial diffusivity and radial diffusivity (RD) images were obtained using DTIFIT from FMRIB Diffusion Toolbox, which fits a diffusion tensor model at each voxel. Voxelwise statistical analysis of the FA data was carried out using Tract-Based Spatial Statistics [22], also part of FSL. All subjects' FA data were aligned into a common space; mean FA image was then created and thinned to create a mean FA skeleton which represents the centres of all tracts common to the group. Each subject's aligned FA data were then projected onto this skeleton and the resulting data fed into voxelwise cross-subject statistics. MD, axial diffusivity and RD maps followed the same procedure and were projected onto the FA skeleton.

### Statistical analysis

We used Fisher’s exact test for categorical variables and Mann-Whitney test for quantitative variables to compare patients with falls and those without falls. Results are presented as mean±SEM or frequencies. Calculations were performed with the SPSS Statistical Package (version 18.0, 2006; SPSS Inc., Chicago, IL). A p value <0.05 was considered statistically significant.

DTI-based voxel-wise statistics included a standard general lineal model and a two-sample t-test unpaired design matrix. To compare FA and non-FA data, a mean FA thresholded skeleton mask was applied through 5000 permutation as recommended. Significant results (Family Wise Error [FEW]) were corrected at p<0.05 using a Threshold-Free Cluster Enhancement method [23]. A correlational analysis of DTI values and neuropsychology was performed using Spearman test.

The sample size was calculated using the program GranMo 7.10 according to previous data on the incidence of cognitive dysfunction as evaluated by the PHES in patients with cirrhosis and falls [6]. Considering an incidence of impaired PHES of 77.3% in patients with falls and 25% in patients without falls, using an alpha error of 0.05 and a power of 0.80, the number of patients needed to detect a significant difference in cognitive dysfunction was 14 in each group.

### Ethics statement

The study protocol conformed to the ethical guidelines of the 1975 Declaration of Helsinki and was approved by the Clinical Research Ethics Committee of Hospital de la Santa Creu i Sant Pau. All patients gave informed consent in writing.

## Results

### Patient’s characteristics

Of the thirty patients included (fifteen with falls and fifteen without falls), 9 were excluded from the analysis of the results because they did not complete the neurological and neuropsychological assessment (one with falls and one without falls), MR was contraindicated (two patients with falls and three without falls), or they developed complications of cirrhosis requiring hospitalization before the end of the study (two patients without falls). Contraindications for MR were claustrophobia in three patients (one with falls and two without falls), a metallic implant in one patient (with falls), and a pacemaker in another patient (without falls). Twenty-one patients were finally analyzed: twelve patients with falls and nine patients without falls. Patients with falls had a total of 26 falls in the 12 months before the study. All falls were accidental at home or in the street. No patient presented falls during an episode of overt hepatic encephalopathy. The injuries resulting from the falls were 20 contusions, 3 wounds and 3 fractures; emergency room care was required in seven patients and hospitalization in one. Table 1 shows that there were no statistically significant differences in clinical and analytical characteristics between patients with falls and patients without falls. We observed a non-statistically significant trend for patients with falls to be slightly older and to present diabetes more frequently than patients without falls.

**Table 1:** Clinical and analytical characteristics of patients with falls and patients without falls.

	**Patients with falls n = 12**	**Patients without falls n = 9**	**p**
Age (yr)	67.5±2.4	63.1±3.7	0.45
Sex (male/female) (%)	4 (33.3)/8 (66.7)	5 (55.6)/4 (44.4)	0.39
Body mass index (kg/m^2^)	26.6±1.6	27.5±1.5	0.82
Educational level (yr)	8.1±1.5	7.1±1.7	0.67
Child-Pugh score	6.2±0.3	6.1±0.2	0.97
MELD ^a^ score	10.2±0.9	11.8±2.8	0.91
Etiology of cirrhosis (%): alcohol/hepatitis C virus/hepatitis B virus	6 (50)/5 (41.7)/1 (8.3)	7 (77.8)/1 (11.1)/1 (11.1)	0.30
Previous decompensations of cirrhosis (%)	8 (66.7)	8 (88.9)	0.33
- Previous ascites (%)	8 (66.7)	8 (88.9)	0.33
- Previous variceal bleeding (%)	1 (8.3)	2 (22.2)	0.55
- Previous encephalopathy (%)	2 (16.7)	0	0.48
Number of previous encephalopathy episodes	0.2±0.1	0	0.55
Present ascites (%)	1 (8.3)	2 (22.2)	0.55
Diuretics (%)	6 (50)	7 (77.8)	0.36
Beta-blockers (%)	7 (58.3)	3 (33.3)	0.38
Nitrates (%)	0	1 (11.1)	0.42
Mean arterial pressure (mm Hg)	90.8±3.9	94.0±2.9	0.69
Orthostatic hypotension (%)	0	0	1.00
Type 2 diabetes (%)	7 (58.3)	3 (33.3)	0.38
Degree of comorbidity ^b^	0.6±0.1	0.4±0.2	0.14
Severe deficit of visual acuity (%) ^c^	2 (16.7)	1 (11.1)	1.00
Hemoglobin (g/L)	124.7±4.9	137.3±5.1	0.19
Serum sodium (mmol/L)	137.3±1.0	138.6±1.4	0.55
Serum albumin (g/L)	34.6±1.4	37.8±2.2	0.14
Serum bilirubin (micromol/L)	19.9±2.6	22.6±3.6	0.62

^a^ Model for end-stage liver disease.

^b^ Modified Charlson index.

^c^ <3/10 using decimal Snellen number chart.

### Neuromuscular assessment

Table 2 summarizes the main findings from the neuromuscular assessment. All patients were conscious and orientated and did not present asterixis. A basic neurological exam showed no relevant alterations in any patients from either of the two groups. There were no significant differences in muscle strength, impaired electroneurography, or scores of ataxia between the two groups. The UPDRS-III score was suggestive of mild subclinical parkinsonism, but there were no significant differences between patients with and without falls.

**Table 2:** Neuromuscular evaluation of patients with falls and patients without falls.

	**Patients with falls n = 12**	**Patients without falls n = 9**	**p**
Impaired muscular strength (%) (MRC) ^a^	0	0	1.00
Impaired electroneurography (%)	2 (16.7)	2 (22.2)	1.00
ICARS ^b^ -I	0.9±0.3	0.6±0.2	0.84
ICARS-II	2.2±0.5	1.5±1.2	0.11
UPDRS-III ^c^	6.4±1.7	4.6±1.7	0.65
Axial subscore of UPDRS-III	1.4±0.4	0.7±0.3	0.31

^a^ MRC: Medical Research Council.

^b^ ICARS: International Cooperative Ataxia Rating Scale.

^c^ UPDRS-III: Unified Parkinson’s Disease Rating Scale-part III.

### Neuropsychological assessment

Table 3 shows patients with falls had a worst performance on PD-CRS (especially in the cortical-posterior score) and PHES. Of the five tests included in the PHES, only Number Connection Tests A and B and the Digit Symbol Test were significantly impaired in patients with falls compared to patients without falls. Patients with falls presented a specific pattern of cognitive impairment compared with patients without falls. This was determined by greater difficulty in executive and visuospatial tasks, as suggested by the statistically significant differences in Rey-Osterrieth Complex Figure Test and WCST subtests.

**Table 3:** Neuropsychological tests in patients with falls and patients without falls.

	**Patients with falls n = 12**	**Patients without falls n = 9**	**p**
MMSE ^a^	25.6±0.9	28.3±0.2	0.06
**PD-CRS** ^b^ total score	69.4±5.4	80.5±3.2	**0.04**
- PD-CRS: Frontal-subcortical	44.0±4.8	52.5±2.7	0.14
- PD-CRS: Cortical-posterior	25.1±0.7	28.0±0.6	**0.01**
**PHES** ^c^	-3.7±0.8	-0.7±0.9	**0.03**
- Number Connection Test-A	105.3±18.9	46.6±6.0	**0.007**
- Number Connection Test-B	275.5±40.8	140.5±22.2	**0.008**
- Digit Symbol Test	16.1±2.5	26.7±4.2	**0.04**
- Serial Dotting Test	55.8±3.7	48.9±4.2	0.26
- Line Tracing Test	132.0±7.4	119.8±14.1	0.37
Impaired PHES (< -4) (%)	7 (58.3)	1 (11.1)	0.06
**Specific neuropsychological tests**			
- Digit Span Forward	4.2±0.2	4.7±0.4	0.42
- Digit Span Backward	2.8±0.2	3.5±0.3	0.12
- Phonetic Fluency	8.2±1.3	10.6±1.4	0.24
- Semantic Fluency	13.0±1.3	16.5±1.0	0.07
- Rey-Osterrieth Complex Figure Test Copy	21.6±2.4	32.2±1.5	**0.003**
- Rey-Osterrieth Complex Figure Test 20 min Recall	6.9±1.6	13.2±2.4	**0.04**
- Iowa Gambling Task Total Score	-2.2±3.0	5.3±5.1	0.19
- WCST ^d^ Corrects	46.4±2.9	62.6±3.9	**0.01**
- WCST Errors	82.3±3.0	66.3±4.0	**0.01**
- WCST Categories	1.4±0.4	3.5±0.7	**0.03**

^a^ Mini-Mental State Examination.

^b^ Parkinson’s Disease-Cognitive Rating Scale.

^c^ PHES: Psychometric Hepatic Encephalopathy Score.

^d^ WCST: Wisconsin Sorting Card Test.

Higher values indicate better results in all tests except for Number Connection Test A and B, Line Tracing Test, Serial Dotting Test and WCST Errors.

### MRI

#### MRI findings

As shown in Table 4, there were no differences in MRI findings (visual assessment) between the two groups.

**Table 4:** Cerebral MRI findings visually assessed in cirrhotic patients with falls and in those without falls.

	**Patients with falls n = 12**	**Patients without falls n = 9**	**p**
T2-hyperintensity in the cerebral white matter (0–2)	1.25±0.15	1.00±0.10	0.25
T2-hyperintensity in the basal ganglia (0–2)	0.33±0.25	0.56±0.20	0.39
T2-hyperintensity in the pons (0–2)	0.33±0.26	0.44±0.27	0.68
Cerebellar atrophy (0–2)	1.33±0.10	1.44±0.21	0.75
Cerebral atrophy (%)	12 (100)	7 (77.8)	0.17
“État criblé” of the basal ganglia (%)	2 (16.7)	2 (22.2)	1.00
Microhemorrhages (%)	6 (50)	5 (55.6)	1.00
T2-hyperintensity of the corticospinal tract (%)	10 (83.3)	5 (55.6)	0.33
Linear T2-hyperintensities along the lateral aspect of the putamina (%)	10 (83.3)	9 (100)	0.48
T1-hyperintensity in the globus pallidus	0.37±0.03	0.39±0.03	0.67
T1-hyperintensity in the putamen	0.27±0.02	0.27±0.02	0.83
GP Index (ratio of T1-hyperintensity in the pallidus to that of the putamen)	1.38±0.03	1.45±0.05	0.33

#### DTI analysis

Using Freesurfer, no difference was found in white matter volumes among groups. However, DTI analysis showed a decrease of fractional anisotropy (FA) and an increase of mean diffusivity (MD) and radial diffusivity (RD) values in patients with falls compared to patients without falls (corrected FWE, significance level of p<0.05). There were no differences between the two groups regarding axial diffusivity.

**Fig 1:**
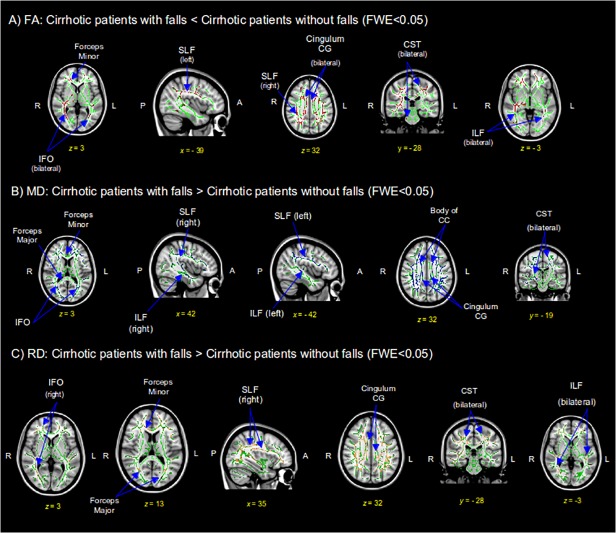
DTI maps show reduced fractional anisotropy (FA) (A) and increased mean diffusivity (MD) (B) and radial diffusivity (RD) (C) in patients with falls compared to those without falls. Results are shown with a Threshold-Free Cluster Enhancement method at p<0.05 corrected. Rows show results of selected coronal, sagital and axial coordenate slices on a MNI152 brain template image (MNI coordinates). Green voxels represent the FMRIB58 white matter skeleton mask. Red voxels have significantly decreased FA values (A), blue voxels imply significantly increased MD (B) and lightbrown voxels represent increased RD values (C). FWE = Family Wise Error; SLF = superior longitudinal fasciculus; CST = corticospinal tract; ILF = inferior longitudinal fasciculus; IFO = inferior frontal-occipital; CC = corpus callosum; CG = cingulate gyrus.

Regarding FA, patients with falls had lower values with a local maximum in the forceps minor (p = 0.01), but also involving both corticospinal tracts (CST), cingulum of the cingulate gyrus (CG), inferior fronto-occipital fasciculus (IFO), inferior longitudinal fasciculus (ILF) and superior longitudinal fasciculus (SLF), bilaterally (Fig. 1A). Patients with falls had increased MD values, the highest difference being located in the body of the corpus callosum (CC) (p = 0.02), but differences were also observed in the forceps minor, forceps major, bilaterally the IFO, ILF, SLF, cingulum of the CG, and the CST (Fig. 1B). Patients with falls also showed increased RD values in the right SLF (p = 0.008), cingulum of the CG, CST and ILF bilaterally, right IFO, forceps major and forceps minor (Fig. 1C). No significant results were found on the reverse contrasts.

**Fig 2:**
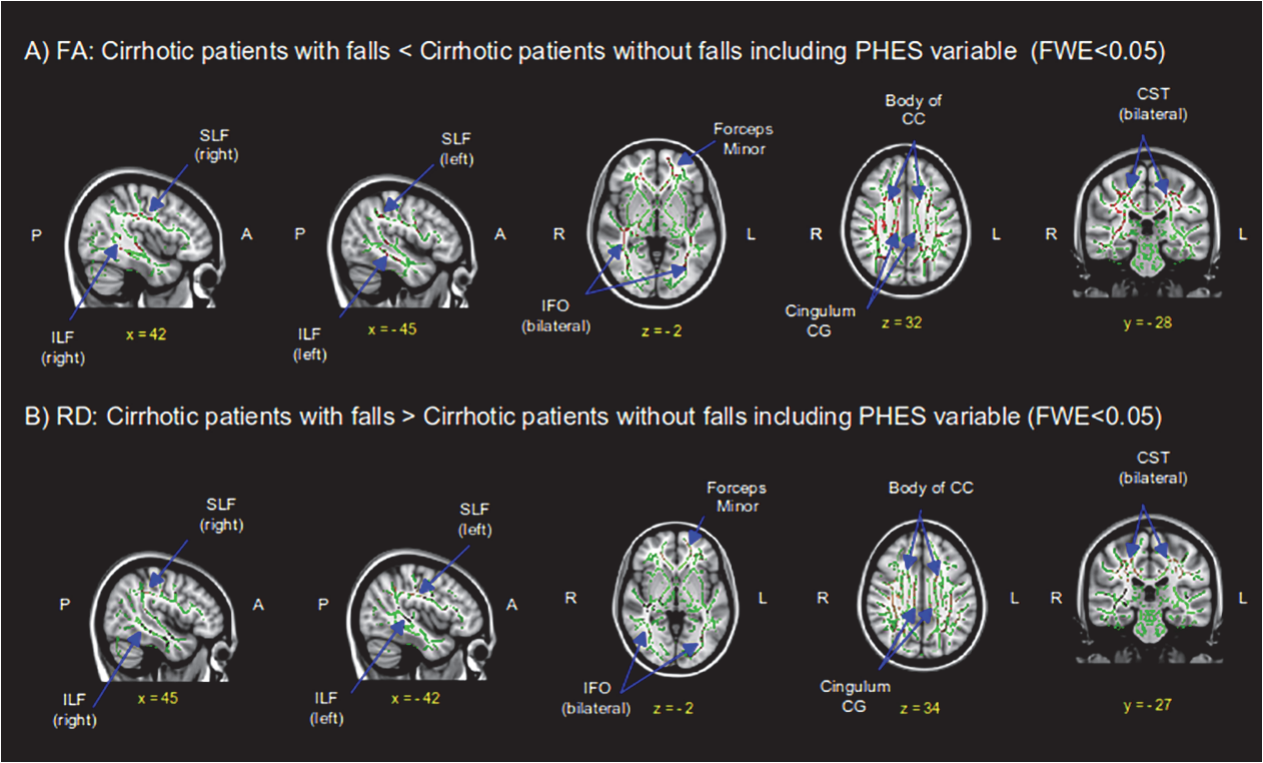
DTI maps show reduced fractional anisotropy (FA) (A) and increased radial diffusivity (RD) (B) in patients with falls compared to those without falls, including Psychometric Hepatic Encephalopathy Score (PHES) as a covariate. Results are shown with a Threshold-Free Cluster Enhancement method at p<0.05 corrected. Rows show selected coronal, sagital and axial maxima coordenate slices on a MNI152 brain template image (MNI coordinates). Red voxels have significantly decreased FA values (A), and brown-lightbrown voxels have significantly increased RD values (B). FWE = Family Wise Error; SLF = superior longitudinal fasciculus; CST = corticospinal tract; ILF = inferior longitudinal fasciculus; IFO = inferior frontal-occipital; CC = corpus callosum; CG = cingulate gyrus.

A second analysis was made including PHES as a covariate, to assess whether white matter abnormalities were independent of PHES (Fig. 2). There was a decrease of FA and an increase of RD values in patients with falls compared to those without falls (p<0.05). FA values were decreased in patients with falls, having a local maxima on the right SLF (p = 0.01), but also involving the left SLF, ILF, IFO, cingulum of the CG and CST bilaterally, forceps minor and body of CC. Additionally, RD values were increased in patients with falls, having a local maxima on the right CST (p = 0.018), but also involving the left CST, body of CC, forceps minor, cingulum of the CG, ILF, IFO and SLF bilaterally. No significant results were found in the reverse contrasts.

#### Correlation between DTI and neuropsychology

**Fig 3:**
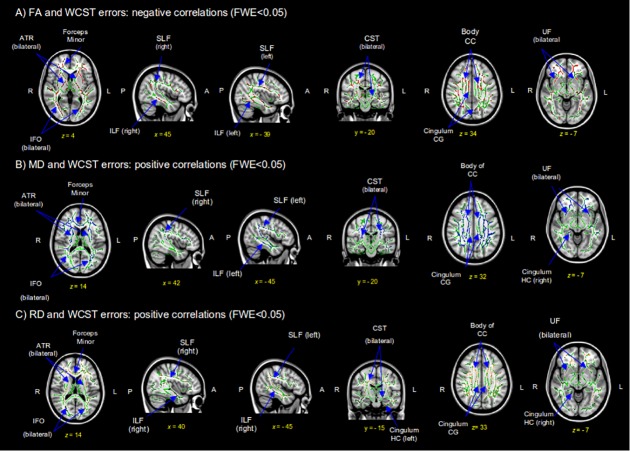
DTI maps show negative fractional anisotropy (FA) (A), and positive mean diffusivity (MD) (B) and radial diffusivity (RD) (C) correlations with Wisconsin Card Sorting Test (WCST) Errors in all cirrhotic patients. Results are shown with a Threshold-Free Cluster Enhancement method at p<0.05 corrected. Rows show selected coronal, sagital and axial maxima coordenate slices on a MNI152 brain template image (MNI coordinates). Red-yellow voxels are negatively correlated FA values (A), blue-lightblue voxels are positively correlated MD values (B) and brown-lightbrown are positively correlated RD values (C) with WCST Errors scores. FWE = Family Wise Error; SLF = superior longitudinal fasciculus; CST = corticospinal tract; ILF = inferior longitudinal fasciculus; IFO = inferior frontal-occipital; CC = corpus callosum; CG = cingulate gyrus; UF = uncinate fasciculus; HC = hippocampus.

We analyzed the correlations between DTI values and neuropsychological scores in all patients. For this analysis we selected the neuropsychological tests more characteristically impaired in patients with falls than in those without falls: PHES, Rey-Osterrieth Complex Figure Test Copy and WCST Errors. Significant correlations were only obtained with WCST Errors (p<0.05). FA values in both anterior thalamic radiations, CST, cingulum of the CG, forceps minor, IFO, ILF, SLF, uncinate fasciculus and body of CC correlated negatively with performance in the WCST Errors (r = -0.840) (Fig. 3A). We also found positive correlations between MD values and WCST Errors scores (p<0.05) in both anterior thalamic radiations, CST, cingulum of the CG, forceps minor, IFO, ILF, SLF, uncinate fasciculus, body of CC and the right cingulum of the hippocampus (r = 0.748) (Fig. 3B). Similarly, RD correlated positively (r = 0.766) with WCST Errors scores (p<0.05) in both anterior thalamic radiations, CST, cingulum of the CG, forceps minor, IFO, ILF, SLF, uncinate fasciculus, body of CC and bilateral cingulum of the hippocampi (Fig. 3C).

## Discussion

The main finding in this study was that patients with cirrhosis and falls presented alterations in cerebral white matter on MR-DTI that correlated with executive dysfunction, compared to patients with cirrhosis without falls.

We did not find any significant differences between these two groups of patients regarding demographic, clinical or analytical parameters that have been previously associated with falling, cognitive dysfunction, or white matter lesions on MRI. These parameters included age, sex, degree of liver insufficiency, previous hepatic encephalopathy, anemia, body mass index, orthostatic hypotension, pharmacological treatment, diabetes, degree of comorbidity, visual acuity, or serum sodium [1,6,8,19,24].

Patients with cirrhosis often present decreased muscle strength and may have peripheral neuropathy caused by alcohol, hepatitis C virus, or diabetes [17,35]. Both factors have been related to an increased risk of falling [8,14]. However, we did not observe any differences in muscle strength or in the incidence of abnormal electroneurography between patients with and without falls. Neither did we find statistical differences in the ataxia scales.

A clear relationship between parkinsonism and falls has been observed in non-cirrhotic patients [10]. Parkinsonism is frequent in patients with cirrhosis, has been associated to cognitive impairment and is considered to be due to alterations in the basal ganglia, mainly manganese deposition [16,36]. Parkinsonian motor features are not only related to basal ganglia alterations, however, but also to dysfunction of the circuits connecting prefrontal and frontal-parietal tracts with the basal ganglia [37]. Disruptions in this network may lead to both cognitive defects and gait disturbances with postural instability evidenced by the axial subscore of UPDRS-III [38], and these two factors could contribute to the higher predisposition to falling in patients with cirrhosis [6,13]. In the present study, we did not observe statistical differences between patients with falls and those without falls regarding MRI alterations of basal ganglia, parkinsonism evaluated by the UPDRS-III total score, and the axial subscore of UPDRS-III. These findings do not therefore support a significant role of parkinsonism in the predisposition to fall in the patients from this study.

Considering neuropsychological testing, patients with falls performed worse in the PHES, a neuropsychological battery used to diagnose minimal hepatic encephalopathy [1,2,31], than patients without falls, as has been previously described [6]. Moreover, the number of patients with impaired PHES (< -4) [31] was higher in patients with falls. The difference did not reach statistical significance, however, probably due to the small sample size. PD-CRS [32], chosen for the previously observed similarities between cognitive dysfunctions in patients with Parkinson’s disease and patients with cirrhosis [6,10,16], was more impaired in those with falls, both for total and cortical-posterior score. When analyzing the specific tasks for cognitive domains, patients with falls presented a pattern of predominant executive and visuospatial dysfunction when compared to patients without falls. All these features are consistent with an impairment in cognitive functions that has been related to falls in populations other than patients with cirrhosis, such as older adults [7,39] and patients with multiple sclerosis [9] or Parkinson’s disease [10].

Considering MRI findings, white matter lesions, cerebral atrophy and cerebellar atrophy have been previously associated with falls in other populations [21,40]. In our study, no differences in visual assessment of these MRI findings were obtained between patients with and without falls.

However, using a more detailed DTI analysis, we found alterations in cerebral white matter integrity in patients with falls compared to those without falls. Specifically, we observed an increase in MD and RD and a decrease in FA in patients with falls. Most previous studies using brain MR-DTI have reported an increase in MD and a decrease in FA when comparing patients with cirrhosis and controls, or patients with hepatic encephalopathy and those without [18,41]. Interestingly, white matter alterations have been related to the degree of liver insufficiency and cognitive dysfunction [19,20,41].

Brain white matter alterations in the MR-DTI of patients with cirrhosis may reflect reversible changes due to low-grade edema [18,19]. This edema is considered the result of water entering astrocytes as an osmotic response to an increase in intracellular glutamine because of ammonia detoxification [19,41]. Inflammation may also play a role in brain edema in these patients by acting synergistically with ammonia [41].

However, white matter alterations in the MR-DTI of these patients may also represent irreversible structural injury [18,19]. Axonal injury and demyelination have been observed at autopsy in patients with chronic hepatic encephalopathy and white matter lesions on MRI [42], and are thought to underlie such irreversible alterations [18,19]. These processes could be due to neurotoxins [19] and/or longstanding edema [42], and would explain how white matter alterations can persist even after liver transplantation [19]. In our study, the increase in MD in patients with falls could suggest either edema or demyelination, but the concomitant increase in RD supports the possible role of the latter [43]. Nevertheless, it should be emphasized that any microstructural features of white matter inferred from DTI maps are indirect measures of the real biological structure, and should thus be interpreted with caution.

In our study, white matter alterations were more marked in the SLF and CST than in other areas. Alterations in these white matter tracts have been associated with specific functional/cognitive disturbances (i.e. executive, visuospatial-visuoconstructive) [15,44–46] that have in turn been related to the predisposition to fall in other populations [7,9,10,39] as well as in the patients from our study. The significant correlation obtained between DTI alterations and executive dysfunction in our study also points in this direction.

It is of note that although patients with falls showed a worse performance in the PHES than patients without falls, as previously described [6], white matter alterations in our study were independent of PHES, as shown by covariate analysis. Chavarria et al. [18] also failed to observe any relationship between white matter alterations and neuropsychological tests. Our findings are in line with the hypothesis that impaired PHES may simply be a surrogate marker of underlying cerebral alterations predisposing patients with cirrhosis to fall [6,13]. The results of the present study suggest that these alterations could imply diffuse impairment in cerebral white matter involving more markedly specific circuits. The dysfunction of these circuits would result in specific cognitive defects that may in turn favour falling. Training addressed to such specific impaired cognitive functions [9] may thus be helpful to decrease the incidence of falls in these patients.

The present study has several limitations. First, the small sample size may have resulted in a type II error in the statistical analysis of some parameters. Second, we can not be sure that the alterations in neuropsychology and cerebral white matter on DTI were present when falls occurred several months before the performance of the study. However, this was a comprehensive study that included complex, non-routinely performed procedures. Therefore, it was difficult to recruit a high number of patients and to perform a prospective study to assess whether the alterations in neuropsychology and DTI were related to falls during follow-up. Third, the strict exclusion criteria might reduce the applicability of the results, but we considered this approach was needed in order to minimize the influence of confounding factors. And finally, there was a non-significant trend for patients with falls to be slightly older and to have a higher incidence of type 2 diabetes than those without falls. Both these factors, in addition to minimal hepatic encephalopathy due to liver disease, may favour cognitive dysfunction, falls and alterations in white matter integrity [8,46,47]. Indeed, cognitive dysfunction is thought to be multifactorial in a high percentage of patients with cirrhosis in our setting [6]. However, we consider this does not invalidate the relationship between cognitive dysfunction, falls and white matter lesions observed in our patients.

In conclusion, although our sample size was small, our results suggest that alterations in cerebral white matter tracts connecting the prefrontal cortex with the posterior parietal cortex are associated with the executive defects involved in the risk of falling in patients with cirrhosis. Further studies are needed to explain the mechanisms involved in these alterations.

### IN MEMORIAM

This article is dedicated to the memory of Juan Cordoba and Manel Barbanoj. We will always remember them for their drive and enthusiasm, their humility and their generosity.
